# Seroprevalence of hepatitis B virus surface antigen (HBsAg) among clients visiting ‘Tefera Hailu’ memorial hospital, Sekota, Northern Ethiopia

**DOI:** 10.1186/s12879-016-1744-3

**Published:** 2016-08-08

**Authors:** Daniel Gebreegziabher, Gebrekidan Gebregzabher Asfeha, Hagos Amare Gebreyesus

**Affiliations:** 1 Medical Laboratory Technology, Institute of Biomedical Sciences, Mekelle University, Mekelle, Ethiopia; 2 Medical biochemistry, Institute of Biomedical Sciences, Mekelle University, Mekelle, Ethiopia

**Keywords:** Hepatitis B virus, Seroprevalence, HBsAg, Tefera Hailu memorial hospital, Liver cirrhosis

## Abstract

**Background:**

Hepatitis B virus is one of the most causative agents of human liver disease, including acute and chronic hepatitis, cirrhosis and hepatocellular carcinoma. The disease is a great health problem worldwide, with estimated of 350 million chronically infected people. Objective: The aim of this study was to determine the prevalence of hepatitis B virus in Tefera Hailu Memorial Hospital (THMH) for the last three years (2013, 2014, 2015) by using secondary data.

**Method:**

A three year retrospective record review was conducted from March 01/2015-July 30/2015. All registered data for hepatitis B virus serological screening of the specified period were included. The data were collected by predesigned data collection sheet. The sample size was calculated by simple statistical estimation to be 149 for each year.

**Result:**

Of 482 subjects, 215 (44.60 %) were females and 267 (55.40 %) were males. The overall prevalence of HBV was 102 (21.16 %). The positivity rate was 69 (14.31 %) in the age group between 15 and 45. There was a decrease in the prevalence of HBV from 2012 up to 2014.

**Conclusion:**

The seroprevalence of HBsAg was higher in males than in females and the yearly prevalence decreases from 2012–2014. But HBV infection is still a public health problem in Ethiopia. Therefore intensification of health education concerning modes of transmission and prevention of HBV, early case finding and treatment is recommended to reduce the spread of the disease.

## Background

Hepatitis B is caused by the hepatitis B virus (HBV), an envelope, double stranded, circular DNA genome and classified within the family Hepadnaviridae. It infects the liver and causes an inflammation called hepatitis, originally known as serum hepatitis. HBV infection can cause acute or chronic illness. The acute illness causes liver inflammation, vomiting, Jaundice and often death. Chronic HBV infection may eventually cause liver cirrhosis and cancer [1–3]. HBV is transmitted parenterally via apparent percutaneous or permucosal exposure to infected blood or other body fluids [4]. In low prevalence areas, HBV is typically a disease of young adults who acquire the infection via mainly unprotected sexual contact or sharing syringes with HBsAg positive people and through exposure to contaminated equipment. In high prevalence regions, most infection occurs prenatally or during early childhood [5].

However, an estimated 3 % to 5 % adults and up to 95 % of children develop chronic HBV infection. Persistent infection can also be either symptomatic or asymptomatic; those with elevated liver chemistries and abnormal biopsies are termed as having chronic hepatitis B and those with normal studies are labeled as chronic HBV carriers. Long term infection increases risk of developing cirrhosis and Hepatocellular carcinoma [6]. Although replication takes place in the liver, the virus spreads to the blood where the virus specific proteins and their corresponding antibodies are found. In infected people blood tests for these proteins and antibodies are used to diagnose the infection [7]. In chronic hepatitis B, the survivals are estimated to be 100 % at 5 years. However, cirrhosis and hematoma are two major long term complications of chronic HBV infection that significantly increase morbidity and mortality [8].

Hepatitis B virus infection remains a major global public health problem. Globally, of the two billion people previously infected, more than 350 million people have developed chronic HBV infection, causing one million HBV related deaths each year [9]. Infection with HBV is highest among developing countries of Asia, Africa, and pacific Islands and lowest among the developed countries or America, Europe and Austria [10]. The majority of chronic carriers of HBV are found in South East Asia and sub-Saharan Africa [11]. Persistence of such HBV infection can lead to chronic hepatitis, cirrhosis and Hepatocellular carcinoma (HCC) [12]. Thus, HBV is a major health problem in developing countries [13, 14].

Infection with HBV is endemic and causes a great public health problem in Africa. In Nigeria for example, 66 % of the population had one or more serological markers of HBV and 9.1 % of them are carriers of HBsAg [15]. In Ethiopia, the overall prevalence of HBV varies from 4.7–16.8 % for HBsAg and 70–76.4 % for at least one marker positive [16]. Similarly a study conducted at JUSH reported a prevalence of 8.2 % [17], indicating HBV infection is of great public health significance in the nation.

HBsAg prevalence among Vietnamese immigrants and refugees in several studies was 10.0 – 16.6 % [18, 19] which reflects 7.4 % for female in the northern territory and 5.7 % for males and 10.0 for females in South Australia. Health care workers are about 20 times more likely to contract the virus than the general public [19–21]. Blood donors were tested in Pakistan for HBsAg and HBV DNA and, 3.41 % were positive for HBsAg by immunochromatographic test, 2.05 % by ELISA and 1.85 % by PCR [22].

In another study conducted in Ethiopia, 72 % were found to have evidence of past or present infection of HBV [23]. Another cross sectional study among healthy blood donors at JUSH showed overall prevalence 24.2 % [24].

In General, hepatitis B virus (HBV) infection and its squelae (cirrhosis and liver cancer) are major global health problems mostly in developing countries including Ethiopia. As already indicated above, the national prevalence of HBsAg is as high as 16 %. Therefore, the aim of this study was to assess the seroprevalence of HBV for the last three years using laboratory recorded data (log book) to fill the existing epidemiologic gap in the area. Moreover, the finding will be used to plan intervention strategies and can serve as baseline information for those who seek to conduct similar studies in the same area or other parts of the country.

## Methods

### Study area

The study was conducted at Tefera Hailu’ Memorial Hospital (THMH) which is located 720 km away from Addis Ababa, the capital city of Ethiopia. Its local temperature is 25–34 °C. The people’s native language is ‘Himra/Agew’ and can speak ‘Amharic’ and ‘Tigrigna’. Currently it is the only hospital in the area giving service to about 521, 127 population in the zone of which 258, 755 are males and the rest 262, 372 are females [25]. On average, there were around 36 thousand annual visits out of which approximately 160 HBsAg tests were ordered per year.

### Study design and period

A retrospective record review of the laboratory logbook was conducted from March 01/2015-July 30/2015.

### Population

All patients visited THMH from January 2012 to Dec 2014 were target and All patients who were screened for HBV and registered in the log book from January 2012-Decemeber 2014 were included in the study.

### Laboratory setttings

HBsAg was ordered as part of the antenatal care panel and for clinical suspicion of liver disease, that is why the prevalence is high. The Wondfo One Step Cassette Style HBsAg test kit (Guangzhou Wondfo Biotech Co. Ltd, China) was being used throughout the study period. The HBsAg test utilized is a One Step Cassette Style which is a rapid, direct binding test for the visual detection of hepatitis B surface antigen (HBsAg) in serum. It is based on the principle of sandwich immunoassay for determination of HBsAg in serum.

### Sampling method

The national prevalence, 10.8 % was considered for sample size (n) determination using the single population proportion formula.

$$ n=\frac{{\left(Z\frac{\alpha }{2}\right)}^2\ P\left(1-P\right)}{d^2} $$

Where:

n = the minimum sample size Zα/2 = 1.966 at 95 % confidence interval

d = margin of error assumed to be (0.05) P = National prevalence rate (10.8 %)

Then:

$$ n=\frac{(1.966)^2\ 0.108\left(1-0.108\right)}{0.05^2} $$

n = 148.9/each year (2012, 2013, 2014)

Even though the minimum sample size was calculated as149/year, to include all patients who visited the hospital during the specified period (January 2012 to Dec 2014) it was elevated to a total of 482.

### Data collection and quality control

A checklist was used to collect data on the status of HBV, age and sex. To ensure the quality of data, the required data was collected from the laboratory log book with due attention to avoid any redundancy. Completeness of the checklist was being asserted through periodic checking.

### Data processing and analysis

The collected date was analyzed using Microsoft Excell following proper entry and cleaning. The result was presented by percentages and explanations using tables and figures.

### Ethical consideration

Written ethical approval was obtained from the research community and ethical review committee of Mekelle University, College of Health Sciences. Moreover, permission was obtained from the Hospital Medical director office.

## Results

A total of 482 HBsAg rapid test records in the laboratory log book were included in this study. Among the 482 study subjects, 215 (44.60 %) were females and 267 (55.40 %) were males.

In 2012, 198 subjects were examined using a marker of HBsAg. 77/198 (38.9 %) were females and 121/198 (61.1 %) were males. In this year, 61/198 (30.8 %) were sero-positive for HBV. The majority of the patients who visited the hospital for HBV screening were in the 15 to 45 age group. Likewise the sero-prevalence of HBV was also higher among this group, 40/198 (20.2 %). With regard to sex, HBV infection was higher in males compared to females, 38/198 (19.2 %) and 23/198 (11.6 %) respectively (Table 1).

**Table 1:** Seroprevalence of HBV in the year 2012 by age and sex of the patients at THMH, Sekota, Northern Ethiopia, 2015

Age in Years	Sex						HBV		Total
	Male			Female					
	Positive	Negative	Total	Positive	Negative	Total	Positive	Negative	
0–14	2 (1.0 %)	12 (6.1 %)	14 (7.1 %)	1 (0.5 %)	4 (2.0 %)	5 (2.5 %)	3 (1.5 %)	16 (8.1 %)	19 (9.6 %)
15-45	25 (12.6 %)	54 (27.3 %)	79 (39.9 %)	15 (7.6 %)	43 (21.7 %)	58 (29.3 %)	40 (20.2 %)	97 (49.0 %)	137 (69.2 %)
>45	11 (5.6 %)	17 (8.6 %)	28 (14.1 %)	7 (3.5 %)	7 (3.5 %)	14 (7.1 %)	18 (9.1 %)	24 (12.1 %)	42 (21.2 %)
Total	38 (19.2 %)	83 (41.9 %)	121 (61.1 %)	23 (11.6 %)	54 (27.3 %)	77 (38.9 %)	61 (30.8 %)	137 (69.2 %)	198 (100 %)

In 2013,134 patients were examined. 58 (43.28 %) were females and 76 (56.71 %) were males. Among the total 134 subjects, 26 (19.40 %) tested positive and the remaining 108 (80.60 %) were negative for HBV. The prevalence of HBV in males was higher than females, 15 (11.19 %) and 11 (8.21 %) respectively (Table 2).

**Table 2:** Seroprevalence of HBV in the year 2013 by age and sex of the patients at THMH, Sekota, Northern Ethiopia, 2015

Age in Years	Sex						HBV		Total
	Male			Female					
	Positive	Negative	Total	Positive	Negative	Total	Positive	Negative	
0–14	2 (1.5 %)	11 (8.2 %)	13 (9.7 %)	3 (2.2 %)	5 (3.7 %)	8 (6.0 %)	5 (3.7 %)	16 (11.9 %)	21 (15.7 %)
15-45	12 (8.9 %)	36 (26.9 %)	48 (35.8 %)	8 (6.0 %)	32 (23.9 %)	40 (29.8 %)	20 (14.93 %)	68 (50.7 %)	88 (65.7 %)
>45	1 (0.7 %)	14 (10.4 %)	15 (11.2 %)	0 (0)	10 (7.5 %)	10 (7.5 %)	1 (0.7 %)	24 (17.9 %)	25 (18.7 %)
Total	15 (11.2 %)	61 (45.5 %)	76 (56.7 %)	11 (8.2 %)	47 (35.1 %)	58 (43.3 %)	26 (19.40 %)	108 (80.6 %)	134 (100 %)

In 2014, 150 subjects were screened. 80 (53.33 %) were females and 70 (46.67 %) were males. Only 15 /150 (10 %) of them were found to be seropositive for HBsAg. As in the previous years, the prevalence of HBV in males was higher than females, 7 (4.67 %) and 8 (5.33 %) respectively (Table 3).

**Table 3:** Seroprevalence of HBV in the year 2014 by age and sex of the patients at THMH, Sekota, Northern Ethiopia, 2015

Age in Years	Sex						HBV		Total
	Male			Female					
	Positive	Negative	Total	Positive	Negative	Total	Positive	Negative	
0–14	3 (2 %)	11 (7.3 %)	14 (9.3 %)	0 (0)	5 (3.3 %)	5 (3.3 %)	3 (2 %)	16 (10.7 %)	19 (12.7 %)
15-45	3 (2 %)	30 (20 %)	33 (22 %)	6 (6 %)	63 (42 %)	69 (46 %)	9 (6 %)	93 (62 %)	102 (68 %)
>45	2 (1.33 %)	−2114 %	23 (15.3 %)	1 (0.7 %)	5 (3.3 %)	6 (4 %)	3 (2 %)	26 (17.3 %)	29 (19.3 %)
Total	8 (5.3 %)	62 (41.3 %)	70 (46.7 %)	7 (4.7 %)	73 (48.7 %)	80 (53.3 %)	15 (10 %)	135 (90 %)	150 (100 %)

The overall sero-prevalence of HBsAg in the years 2012 to 2014 was 21.2 %. The sero-prevalence in males was higher than in females, 22.8 % and 19.1 % respectively. Out of the HBV infected patients, 59.8 % were males and 40.2 % were females (Table 4).

**Table 4:** Seroprevalence of HBV from Jan 2012 – Dec 2014 in terms of sex of the patients at THMH, Sekota, Northern Ethiopia, 2015

Year	Sex						HBV		Total
	Male			Female					
	Positive	Negative	Total	Positive	Negative	Total	Positive	Negative	
2012	38 (19.2 %)	83 (41.9 %)	121 (61.1 %)	23 (11.6 %)	54 (27.3 %)	77 (38.9 %)	61 (30.8 %)	137 (69.2 %	198 (100 %)
2013	15 (11.2 %)	61 (45.5 %)	76 (56.7 %)	11 (8.2 %)	47 (35.1 %)	58 (43.3 %)	26 (19.4 %)	108 (80.6 %)	134 (100 %)
2014	8 (5.3 %)	62 (41.3 %)	70 (46.7 %)	7 (4.8 %)	73 (48.7 %)	80 (53.33 %)	15 (10 %)	135 (90 %)	150 (100 %)
Total	61 (22.8 %)	206 (77.2 %)	267 (100 %)	41 (19.1 %)	174 (80.9 %)	215 (100 %)	102 (21.2 %)	380 (78.8 %)	482 (100 %)

**Fig. 1:**
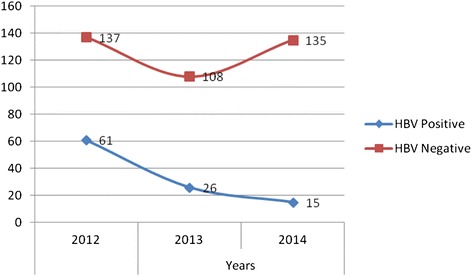
Three years trend of seroprevalence of HBV from Jan 2012 – Dec 2014 at THMH, Sekota, North Ethiopia, 2015

Regarding the age of the study subjects in all years of study, 67.8 % were in the age group of 15-45 years. Moreover the sero-prevalence of HBV was also comparatively higher among this age group, 14.3 % (Table 5). There is a general decrease in the seroprevalence of HBV from 30.8 % to 10 % for the last three years (Fig. 1).

**Table 5:** Seroprevalence of HBV from Jan 2012-Dec 2004 in terms of age group the patients at THMH, Sekota, Northern Ethiopia, 2015

Age in Years	Sex						HBV		Total
	Male			Female					
	Positive	Negative	Total	Positive	Negative	Total	Positive	Negative	
0–14	7 (1.4 %)	34 (7.1 %)	41 (8.5 %)	4 (0.8 %)	14 (2.90 %)	18 (3.7 %)	11 (2.3 %)	48 (9.9 %)	59 (12.2 %)
15-45	40 (8.3 %)	120 (24.9 %)	160 (33.2 %)	29 (6.0 %)	138 (28.6 %)	167 (34.6 %)	69 (14.3 %)	258 (53.5 %)	327 (67.8 %)
>45	14 (2.9 %)	52 (10.8 %)	66 (13.7 %)	8 (1.6 %)	22 (4.6 %)	30 (6.2 %)	22 (4.7 %)	74 (13.3 %)	96 (19.9 %)
Total	61 (12.7 %)	206 (42.7 %)	267 (55.4 %)	41 (8.5 %)	174 (36.1 %)	215 (44.6)	102 (21.2 %)	380 (78.8 %)	482 (100 %)

## Discussion

The limitations of the majority of serological and DNA tests were also considerable to the test kit used by this study. Determination of HBsAg alone may not exactly measure the overall prevalence of HBV infection in a given population as other viral co-infections such as HIV or HCV are indicators of the total infection rate. The carrier state of HBsAg is defined as persistence of this antigen in the blood for over 6 months. Thus, a single determination of HBsAg may not be the ideal way of defining the carrier state among study subjects.

Keeping the limitations, the overall seroprevalence of HBsAg in this study was 21.2 %% of which 12.7 % were males and 8.5 % were females. This finding was lower than reports from Logos, Nigeria, 25.7 % [12], and Jimma, Ethiopia, 24.2 % [22]. However, our finding was higher than reports from countries like in Central Africa Republic 15.5 % [26], Kenya 8.8 % [27], Nigeria 7.1 % [28], and two studies in India reporting, 4.9 % [29] and 0.61 % [30]. Moreover it was higher than two prevalences 3.4 % [21] and 9.8 % from Pakistan [31], and one Palestin syudy 8.1 % [32]. This difference may be due to geographical variation, genetic difference; immunity, other socioeconomic characteristics and sample size variation also may contribute to this difference.

The finding of this study was also higher than any of the cross-sectional studies conducted in Ethiopia, Addis Ababa, 7 % [23], Southwest, 3.7 % [33], and Northern, 6.2 % [34]. All are lower than our finding due to may be sample size difference, geographical variation, method of detection or this study was done on suspected patients, that increase rate of positivity.

The sex based seroprevalence of HBsAg was higher in males-12.7 % than in females-8.5 %. This is in line with reports from Pakistan −12.7 % in males and 5.8 % in females [22], from Jimma University, Ethiopia – 22.6 % in males and 1.6 % in females [24], from Palestine – 6.5 % in males and 1.6 % in females [32]. In contrast a study from Kenya reported that the prevalence was higher in females 9.4 % [27] while another study in Ethiopia reported no difference among males and females [16].

The year to year decrement in the prevalence of HBsAg observed in our study may indicate time to time increment in awareness in the community and advancement of Health services. However; one study showed that hepatitis medications are not affordable to the majority of the population and the situation is not changed. Since there is no public funding/subsidy for hepatitis infections in Ethiopia, payment is generally out-of-pocket by the individual. Some anti-retroviral drugs given for HIV act against HBV but the government policy in Ethiopia restricts the use of these drugs for the treatment of HBV for fear of the emergence of drug resistance. Hence, for mono-infected cases of chronic HBV, the drugs (tenofovir, lamivudine) are not readily available either in the public or private sectors in Ethiopia. However, HIV–HBV co-infected patients receive free treatment at HIV clinics [35].

Therefore, further investigation of HBV with other coifections and possible risk factors using large sample with a longer period of study involving control groups should be carried out in order to get more ideal information.

## Conclusion

Infection induced hepatitis is a severe cause of mortality and morbidity especially in developing countries. In this study the seroprevalence of HBsAg was 21.5 % and it was higher in males than in females. The yearly prevalence decreases from 2012–2014. However; the study indicates that HBV infection is still a public health problem which should be among the prioritized health problems in the country.

## Abbreviations

DNA, deoxyribonucleic acid; HBsAg, hepatitis B surface antigen; HBV, hepatitis B virus; HCV, hepatitis C virus; HIV, human immune deficiency virus; JUSH, Jimma University Specialized Hospital; THMH, Tefera hailu memorial hospital
